# Anemia and malnutrition in geriatric hospitalized patients: a cross-sectional retrospective study

**DOI:** 10.1186/s12877-025-06287-9

**Published:** 2025-08-20

**Authors:** Elisabeth Lucia Zeilinger, Bärbel Sturtzel, Alexa Leonie Meyer, Jakob Pietschnig, Caterina Sturtzel, Julia Lehner, Chiara Popinger, Gerald Ohrenberger, Ibrahim Elmadfa, Matthias Unseld

**Affiliations:** 1 Department of Clinical Research SBG, Academy for Ageing Research, Haus der Barmherzigkeit, Vienna, Austria; 2 Department of Clinical and Health Psychology, Faculty of Psychology, University of Vienna, Liebiggasse 5, A-1010, Vienna, Austria; 3 Department of Developmental and Educational Psychology, Faculty of Psychology, University of Vienna, Vienna, Austria; 4 Innovative Cancer Models, St. Anna Children’s Cancer Research Institute, Vienna, Austria; 5 Department of Nutritional Sciences, Faculty of Life Sciences, University of Vienna, Vienna, Austria

**Keywords:** Anemia, Geriatrics, Malnutrition, Nutrients, Inflammation

## Abstract

**Background:**

Nutritional factors contributing to anemia in older adults are in need of clarification. We investigated associations between nutritional biomarkers and the incidence of anemia.

**Methods:**

A cross-sectional study was conducted in two centers. Data were collected from patients living in long-term care hospitals. The Geriatric Nutritional Risk Index (GNRI) was applied to determine nutritional risk. Blood parameters were obtained from medical records. Anemics vs. non-anemics were assigned according to hemoglobin levels following the WHO guidelines. Multiple linear regression analysis were performed for statistical analysis.

**Results:**

The sample consisted of *N* = 97 geriatric patients (mean age 84.9 years, 86% female). Anemic patients had a significantly lower GNRI (*M* = 90.6 ± 5.94; *p* =.007) than non-anemic patients (*M* = 94.7 ± 6.11). Serum albumin (*p* =.008), blood iron (*p* <.001), number of erythrocytes (*p* <.001), and the hematocrit value (*p* <.001) were also significantly lower in patients with anemia. Multiple linear regression showed that serum albumin concentration, in addition to the hematocrit, was the driving factor for hemoglobin concentration in anemic patients (*p* =.004; *R*²=0.77).

**Conclusion:**

The present study indicates that nutritional risk plays a substantial role in anemia development in older adults. These findings may be attributable to multifactorial metabolic pathways of macro- and micronutrients on blood hemoglobin concentration. Malnourished geriatric patients with anemia may benefit from a diet rich in protein and iron-rich foods.

## Introduction

A healthy diet has a positive impact on health throughout the life cycle. Epidemiological studies and clinical trials suggest that the daily diet can influence overall health and help prevent common non-communicable diseases such as cardiovascular disease, diabetes and obesity, which are among the leading causes of death and disability worldwide [1, 2].

Anemia and malnutrition have a high incidence among geriatric patients, and both conditions are associated with adverse clinical outcomes [3]. The question of whether geriatric malnutrition serves as an underlying cause of anemia has been a subject of discussion, particularly in light of established associations between anemia and hypoalbuminemia observed in hospitalized geriatric patients [3, 4]. Presently, deficiencies in micronutrients such as iron, folate, and B12 remain the primary focus of investigations concerning determinants of anemia development [5–7]. The present study used a comprehensive approach to malnutrition, the Geriatric Nutritional Risk Index (GNRI) [8], and investigated whether there are differences in nutrition-dependent indicators between anemic and non-anemic geriatric patients.

A well-balanced diet entails consuming macronutrients in appropriate proportions to fulfill energy and physiological needs without excess, while also ensuring an adequate supply of micronutrients and hydration to sustain body metabolism and cellular activities [1]. The pivotal role of a well-balanced diet in promoting healthy aging is substantiated by numerous epidemiological studies, clinical trials, and the World Health Organization’s (WHO) Global Action Plan for the Prevention and Control of Noncommunicable Diseases 2013–2020 [2]. The WHO Global Action Plan seeks to guide daily food choices to impact health positively, endorse the prevention of prevalent non-communicable diseases, and prevent malnutrition [1, 2].

Malnutrition, defined as an imbalance in nutrient intake, whether deficient or excessive, or impaired nutrient utilization, is alleviated by a well-balanced diet [2]. Only a well-balanced diet can ensure that individual cells, such as erythrocytes, synchronize their growth rate or proliferation with nutrient availability while maintaining overall body metabolism [9]. Macronutrients, encompassing carbohydrates, proteins, and fats, supply the energy essential for daily cellular processes, while micronutrients, namely vitamins and minerals, are required in smaller quantities for normal growth, development, metabolism, and physiological functioning [1, 10].

Malnutrition is often associated with a lack of red blood cells and the development of anemia [11, 12]. Anemia is defined as a deficiency in red blood cells or in the concentration of hemoglobin in the blood. Iron, vitamin B12 and folic acid deficiencies are among the most discussed nutritional factors that may contribute to the pathogenesis of anemia [5–7]. However, geriatric malnutrition and deficiencies of specific nutrients, such as the macronutrient protein, may also contribute to the development of nutritional anemia [11, 12]. However, whether and how protein deficiency or geriatric malnutrition in general may be associated with the development of anemia remains to be determined and requires further exploration [4, 6, 7]. Understanding the complex relationship between protein deficiency, geriatric malnutrition and the occurrence of anemia is essential to a comprehensive understanding of the multiple factors influencing nutritional health in the geriatric patients.

It has been suggested that the role of protein in erythropoiesis may be responsible for frequently observed anemia occurrences in cases of protein-energy-malnutrition [6, 13]. However, the interaction of the micronutrients iron, folic acid and B12 and the macronutrient protein with geriatric malnutrition has not yet been sufficiently researched. Experimental evidence showed that low-protein diet-based malnutrition in mice leads to lower numbers in red blood cells and hemoglobin concentration compared to controls [13]. Findings from epidemiological and clinical studies in humans are consistent with these results and show a link between chronic anemia and low albumin concentrations [3, 4, 14], without, however, taking into account the interaction of nutrients.

The risk and incidence of malnutrition increases with age. Physiological changes and the accumulation of chronic diseases or frailty syndromes can alter dietary habits, might misbalance the nutrient uptake, and promote nutrient deficiencies and the onset of malnutrition [10, 15–17]. A multitude of factors are involved in this process, such as various lifestyle factors, diseases and general ageing processes, and often a progressive negative interaction between these factors is observed [17]. At present, malnutrition represents a major health concern in older patients and is associated with negative health outcomes [18] and several syndromes, such as frailty [19], sarcopenia [20], or inflammation [16, 21, 22].

The multifactorial condition of geriatric malnutrition is not comprehensively defined, making a clear diagnosis difficult [17, 23]. Body weight, weight loss or albuminemia are often used as single parameters to define geriatric malnutrition [17]. However, despite their frequent use, these single parameters are not suitable for the reliable detection of geriatric malnutrition. For example, albuminemia remains an unreliable indicator of nutritional status because it may actually be more related to inflammation or hydration status than to malnutrition [8]. As there is a consensus that single parameters such as anthropometric measures or biomarkers do not capture the complex nature of malnutrition [23, 24], there is high demand for a more comprehensive and refined method which integrates multiple measurements. According to the Global Leadership Initiative on Malnutrition (GLIM), a comprehensive malnutrition diagnosis requires a two-step approach: initial risk screening followed by a diagnostic assessment based on phenotypic and etiological criteria [24]. The present study focuses on nutritional risk assessment using the Geriatric Nutritional Risk Index (GNRI). The GNRI is a comprehensive approach that quantifies the nutritional risk of geriatric patients by utilizing both anthropometric measurements and serum albumin concentration [8]. In the present study, we aimed to investigate the association between anemia and nutritional risk in a hospitalized geriatric population.

Our objectives are (1) to examine the extent of anemia and nutritional risk in a geriatric hospitalized sample, and (2) to examine whether there are differences in nutrition-dependent indicators between anemic and non-anemic geriatric patients.

## Materials and methods

### Study design and participants

This study was a cross-sectional retrospective study in two-long-term care hospitals in Vienna, Austria. The final sample consisted of 97 inpatients. A total of 84 (86%) patients were female. The mean age of the sample was 84.9 years (*SD* = 7.9). All patients in the study required not only assistance on a daily basis, but also ongoing medical care to address their complex health needs. The inclusion criteria were: (1) being a hospitalized geriatric patient at one of the two research sites, and (2) having complete data on blood parameters and the GNRI, (3) informed consent was provided by the patient and/or the legal guardian. Prior to inclusion, all participants provided written informed consent. In cases where patients had a legal guardian overseeing their affairs, additional consent was sought from the legal guardian. Data were first pseudonymized during data collection. A key file linking the data to the patient record was stored in a password-protected file and accessible only to the persons collecting the data. Once the final data set was obtained, the data were anonymized. The research received ethical approval from the City of Vienna’s ethics committee (EK-13-043-0513).

### Clinical assessments

The blood parameters used in this study were obtained from regular patient records. The following blood parameters were included in the study: hemoglobin, erythrocytes, mean corpuscular volume (MCV), mean cell hemoglobin concentration (MCH), mean corpuscular hemoglobin concentration (MCHC), hematocrit, free iron, transferrin, ferritin, albumin, C-reactive protein (CRP), folic acid and vitamin B12. These blood parameters were selected because of their potential relationship with nutritional status and/or anemia.

Albumin is related to malnutrition and nutritional risk in hospitalized patients, and is one of the most widely used and cost-effective nutritional markers for the assessment of patients at risk of malnutrition during hospitalization [25, 26]. In general, reduced albumin levels are often observed in older adults, especially those living in institutions, and a gradual decline in serum albumin concentration may be associated with the ageing process [27, 28].

Elevated CRP levels represent inflammation and can be linked to and be a risk factor for age-related diseases and syndromes such as frailty or cardiovascular disease. Studies have also shown a clear link between diet and inflammation [21, 29]. In general, CRP levels between 0.5 and 3.0 mg/dL are classified as mild inflammation, and CRP levels above 3.0 mg/dL are classified as moderate to severe inflammation [29]. Furthermore, when assessing the overall nutritional status of geriatric patients in institutional settings, it is suggested that transferrin levels also be considered, as they may act as surrogate markers of protein deficiency [30].

In addition, the body weight of each patient was recorded. All parameters used in this study are listed in Table 1. Patients were divided into anemic and non-anemic subsamples according to current the WHO guidelines for the assessment of anemia (hemoglobin < 12 g/dl for women and < 13 g/dl for men) [31].

**Table 1:** Clinical parameters for the total sample and subdivided into anemic and non-anemic patients

Parameter	Reference^1^	(mean ± SD) All patients (*N* = 97)	Non-anemic (*n* = 69)	Anemic (*n* = 28)	*p*-value
Hemoglobin (g/dl)	12.00–16.00	12.79 (± 1.32)	13.40 (± 1.01)	11.28 (± 0.77)	**< 0.001**
GNRI	> 98	93.5 (6.32)	94.7 (± 6.11)	90.6 (± 5.94)	**.007**
Erythrocytes (T/l)	3.80–5.20	4.42 (± 0.45)	4.59 (± 0.33)	4.02 (± 0.45)	**< 0.001**
MCV (fl.)	78.00–98.00	88.94 (± 5.54)	89.44 (± 4.23)	87.71 (± 7.87)	0.082
MCH (pg)	27.00–33.00	28.96 (± 2.10)	29.22 (± 1.60)	28.33 (± 2.93)	0.061
MCHC (g/dl)	31.50–36.00	32.56 (± 1.00)	32.68 (± 0.86)	32.26 (± 1.25)	0.123
Hematocrit (%)	35.00–47.00	39.33 (± 3.93)	41.11 (± 3.05)	35.03 (± 2.02)	**< 0.001**
Free iron (µg/dl)	37.00-145.00	70.55 (± 26.2)	76.37 (± 27.2)	56.51 (± 19.0)	**< 0.001**
Transferrin (mg/dl)	200.00-310.00	219.0 (± 50.6)	221.3 (± 41.3)	213.3 (± 66.1)	0.171
Transferrin %	16.00–45.00	24.2 (± 10.1)	25.4 (± 09.9)	21.4 (± 10.1)	0.088
Ferritin (ng/ml)	15.00–150.00	152.4 (± 142.3)	157.3(± 145.3)	140.4 (± 136.9)	0.580
Albumin (g/dl)	3.35–5.29	3.54 (± 0.41)	3.62 (± 0.39)	3.37 (± 0.39)	**0.008**
Body weight (kg)	-	67.04 (± 15.7)	67.84 (± 15.7)	65.07 (± 15.9)	0.306
CRP (mg/dl)	< 0.5	0.84 (± 1.27)	0.79 (± 1.30)	0.96 (± 1.21)	0.219
Folic acid (ng/ml)	3.10–20.50	6.53 (± 3.87)	6.90 (± 4.28)	5.65 (± 2.44)	0.264
Vit. B 12 (pg/ml)	191.0–663.0	391.5 (± 222.5)	408.6 (± 249.6)	349.5(± 129.9)	0.348
BMI	26.79 (± 5.58)		27.12 (± 5.64)	25.97 (± 5.43)	0.257

The table shows the means and standard deviations of the clinical parameters once for the total sample and separately for the anemic and non-anemic groups. The results of Mann-Whitney U tests are given in the last column. Significant results at the 5% level are shown in bold

*MCV* Mean corpuscular volume, *MCH* Mean cell hemoglobin concentration, *MCHC* Mean corpuscular hemoglobin concentration, *GNRI* Geriatric Nutritional Risk Index, *CRP* C-reactive protein, *BMI* Body mass index

¹ standard clinical laboratory references

The nutrition related clinical risk of the patients was assessed by means of the Geriatric Nutritional Risk Index [GNRI; 8]. The GNRI is a clinical, biological index that assesses nutritional risk in geriatric patients. It was proposed by Bouillanne and colleagues [8] to specifically assess nutritional risk in geriatric patients. The GNRI is calculated as follows: GNRI= [1.489 x albumin (grams/L)] + [41.7 x (weight/ideal body weight)]. The usual cut-off for identifying nutritional risk by using the GNRI is 98 [8].

### Statistical analysis

First, we used a Mann-Whitney U test to examine differences between the anemic and non-anemic sample in terms of nutritional risk, using the GNRI as indicator. Second, we explored differences between the anemic and non-anemic sample in terms of blood parameters, also using Mann-Whitney U tests. Significance level for these analyses was set to 5%. These tests were conducted as exploratory analyses to identify potentially relevant variables for inclusion in the regression models. Due to the limited sample size and the risk of increasing Type II error, no correction for multiple testing (e.g., Bonferroni adjustment) was applied.

As a final step in our analysis, three multiple linear regression analysis with hemoglobin concentration as the outcome variable were calculated. We examined the total sample as well as the sub-sample with anemia and the sub-sample without anemia. The individual blood parameters that exhibited significant results in the exploratory Mann-Whitney U tests were entered as predictors. These predictors included erythrocyte count, hematocrit, free iron and albumin concentration. By including these parameters in the regression model, we aimed to uncover their collective effects on hemoglobin concentration in all three samples, in the total study population as well as in the anemic and the non-anemic groups. The GNRI was excluded as a predictor from the regression analysis as it is based on serum albumin concentration and would have resulted in direct dependent variables in the regression model. Variance Inflation Factors (VIFs) were examined. VIF values greater than 5 indicate inflated variance of the estimated regression coefficients due to the multicollinearity among the predictors [32, 33]. In this case, the individual impact of each predictor and the calculation of the coefficient estimates would be compromised. The examination of VIFs in the present analysis indicated no multicollinearity between predictors (see Table 2). Data was analyzed using SPSS v. 24.0.

**Table 2:** Linear regression models investigating predictors of hemoglobin concentration

	Model I: total sample							Model II: no anemia							Model III: anemia					
	Estimate	SE	95% CI		*p*	VIF		Estimate	SE	95% CI		*p*	VIF		Estimate	SE	95% CI		*p*	VIF
			LL	UL						LL	UL						LL	UL		
Intercept	0.167	0.484	−0.793	1.128	0.730			0.205	0.715	−1.224	1.634	0.776			1.745	1.42	−1.185	4.676	0.231	
Erythrocytes (T/l)	− 0.098	0.164	−0.424	0.229	0.554	3.612		− 0.05	0.225	−0.499	0.398	0.823	3.053		− 0.115	0.267	−0.665	0.436	0.671	2.533
Hematocrit (%)	0.336	0.020	0.296	0.376	**< 0.001**	3.954		0.316	0.025	0.266	0.366	**< 0.001**	3.001		0.332	0.055	0.219	0.446	**< 0.001**	2.165
Free iron (µg/dl)	0.004	0.002	0.001	0.008	**0.025**	1.418		0.002	0.002	−0.002	0.006	0.252	1.293		0.009	0.005	−0.001	0.019	0.088	1.516
Albumin (g/dl)	− 0.127	0.105	−0.335	0.081	0.230	1.205		0.076	0.118	−0.159	0.311	0.523	1.147		− 0.63	0.195	−1.034	−0.227	**.004**	1.058
*R* ^*2*^	0.92							0.88							0.77					
*p*	< 0.001							< 0.001							< 0.001					
*N*	97							69												

*SE* Standard Error, *CI* Confidence Interval, *VIF* Variance Inflation Factor

Significant results at the 5% level are shown in bold

### Sample size considerations

This retrospective study included all eligible patients from two long-term care hospitals, resulting in a final sample of 97 geriatric inpatients. A post hoc sensitivity analysis using G*Power indicated that for group comparisons (anemic vs. non-anemic), the study had 80% power to detect medium effect sizes at α = 0.05. For the multiple linear regression models with four predictors, the sample size was sufficient to detect small-to-medium effect sizes. However, the limited subgroup size may reduce power for detecting smaller effects.

## Results

The mean hemoglobin concentration in the sample was 12.79 g/dl (*SD* = 1.32), which is at the lower end of the normal reference range (anemia hemoglobin < 12 g/dl for women and < 13 g/dl for men). According to the WHO guidelines for the assessment of anemia, 29% (*n* = 28) of the patients were anemic and 71% (*n* = 69) were non-anemic, presenting with mean hemoglobin concentrations of 11.28 g/dl (*SD* = 0.77) and 13.4 g/dl (*SD* = 1.01), respectively.

To contextualize this in terms of potential malnutrition, the GNRI, an assessment of nutritional risk, was determined using the parameters of albumin and body weight as specified in the methods section. The overall mean GNRI for the total sample was 93.5 (*SD* = 6.32), which is lower than the recommended value of 98 [7] and indicates a general tendency of nutritional risk in the sample. To further analyze a possible link between anemia and nutritional risk in geriatric patients, the GNRI was compared between anemic and non-anemic patients. The anemic subsample had a markedly lower GNRI than the non-anemic subsample, with 90.6 (*SD* = 5.94) and 94.7 (*SD* = 6.11), respectively (see also Table 1). This difference was found to be statistically significant (*p* =.007), indicating greater nutritional risk in the anemic sub-sample.

In addition, other blood parameters that may be involved in the development of the observed anemia, were compared between the anemic and non-anemic group. The overall mean albumin concentration of the entire sample population was found to be 3.54 g/dl, which is at the lower end of the established reference range. On closer examination of the subgroups within the sample, the non-anemic subgroup had a higher mean albumin concentration of 3.62 g/dl. Conversely, the anemic subgroup had a markedly lower mean albumin concentration of 3.37 g/dl, which is just within the accepted normal range. This difference between the anemic and non-anemic groups was found to be statistically significant at *p* =.008, indicating a lower albumin level and a higher risk of nutritional risk in the anemic subsample.

Our analysis shows, that the anemic patients also had significantly lower concentrations in the blood serum of free iron (*p* <.001), the number of erythrocytes (*p* <.001), and the hematocrit value (*p* <.001). No significant differences were found in relation to CRP levels, the vitamin B_12_ and folic acid serum concentrations, or in the concentrations of the iron metabolism parameters (transferrin, transferrin percentage and ferritin). The mean corpuscular volume (MCV), mean cell hemoglobin concentration (MCH), as well as the mean corpuscular hemoglobin concentration (MCHC) did not show a significant difference between the two groups either (see Table 1).

In the multiple regression analysis, the total population’s hemoglobin concentration could be explained primarily by the hematocrit value (*p* <.001) and the free iron concentration in the blood (*p* =.025). A total of 92% variance could be explained by this model. In the non-anemic patients, the hemoglobin level was explicable by the hematocrit, only (*p* <.001*, *R*^2^ = 0.88). The regression model on the anemic patients *(R*^*2*^ = 0.77), however, showed that the hemoglobin concentration could be explained by the hematocrit value (*p* <.001) and the albumin level (*p* =.004*), indicating a significant impact of nutrition related parameters on the hemoglobin levels in the anemic subsample. All three models were significant with *p* <.001. Detailed results of these models are given in Table 2.

## Discussion

Our findings show that elevated nutritional risk may contribute to anemia onset in geriatric patients. Although both anemic and non-anemic groups in our study were malnourished (i.e., neither group showed a mean GNRI well above 98), the anemic geriatric patients were significantly more malnourished than the non-anemic ones.

Vitamin B12 and folic acid are important cofactors in erythropoiesis and are commonly associated with nutritional anemia in older adults [6, 11]. However, our study did not find significant differences in their serum concentrations between the anemic and non-anemic groups. Both groups exhibited mean levels within the normal reference range. This suggests that, in our sample, vitamin B12 and folate deficiencies were unlikely to be primary contributors to anemia. Nevertheless, given their established role in red blood cell formation [6, 34] and vulnerability to age-related malabsorption, particularly in the presence of atrophic gastritis or certain medications [11, 35, 36], these micronutrients remain important in the context of geriatric anemia. While not directly implicated in our findings, monitoring these nutrients remains important in clinical practice.

Although CRP was measured as an indicator of systemic inflammation, no significant differences were observed between the anemic and non-anemic groups. However, elevated mean CRP levels were observed in both groups, suggesting a low-grade inflammatory burden across the entire sample, likely reflective of chronic comorbidities common in geriatric inpatients. This may have masked any group-specific differences, limiting our ability to isolate the specific role of inflammation in anemia development. Nevertheless, inflammation is a well-established contributor to anemia of chronic disease via mechanisms such as impaired iron utilization and hepcidin-mediated sequestration of iron stores [37–40]. Our findings are consistent with prior studies showing that CRP levels may remain uniformly elevated in institutionalized older adults, making it difficult to use CRP alone as a distinguishing factor for anemia etiology [21].

In line with this, mean MCV values did not significantly differ between anemic and non-anemic patients and were within the normal (normocytic) range in both groups. This suggests that the anemia in our sample does not align with typical patterns of microcytic anemia (often associated with iron deficiency) or macrocytic anemia (typically related to vitamin B12 or folate deficiency) [41]. Instead, the normocytic profile may point toward anemia of chronic disease (also called anemia of inflammation), which is frequently normocytic and multifactorial in etiology [40]. This form of anemia is commonly seen in older adults with chronic comorbidities, where iron metabolism, erythropoiesis, and nutrient availability may all be subtly impaired [42]. Thus, the combination of normocytic anemia and elevated CRP levels across both groups points toward a complex interplay of inflammation and nutritional risk in contributing to anemia in geriatric patients.

The multiple linear regression analysis showed that in anemic patients, the serum albumin concentration and the hematocrit value were the decisive predictors of the hemoglobin level. When considering serum albumin concentration to represent a surrogate marker for the nutritional-and protein-status, these findings suggest that the nutritional status of the anemic patients likely triggered the anemia, particularly because it seems that in the anemic group, the blood iron concentration played only a minor role in the development of the anemia. In contrast, albumin was not a significant predictor in the total sample. This discrepancy may be explained by the dominant influence of hematocrit and iron status across the full cohort, which likely overshadowed the more subtle effect of nutritional status. The subgroup analysis in anemic patients, who may already have depleted iron stores, allowed the nuanced role of protein-related nutritional status to emerge more clearly.

If we equate geriatric malnutrition with protein-energy malnutrition, it would seem obvious that a disrupted protein-energy metabolism could be responsible for these observations. When dietary protein is a source of energy as well as amino acids, protein-energy malnutrition results in reduced protein availability and impaired protein metabolism. Proteins are macromolecules made up of amino acids (AAs). In well-nourished people, protein synthesis and breakdown are in a well-balanced equilibrium. This is not the case in malnourished people. As a result, the hemoglobin molecule cannot be built efficiently enough. Normally, it is assembled in a very differentiated way in the erythrocyte. In order to transport the demanding and essential molecule oxygen, amino acids and iron must be assembled according to the energy status of the cell [9, 15]. The bioavailability of iron must be coordinated with the bioavailability of amino acids for the assembly of the hemoglobin tetramer, which consists of four globin subunits. These conditions affect the translational process, the entire biochemical synthesis, and cause faulty erythropoiesis in malnourished people due to deficiencies and imbalances in nutritional input, leading to a reduced number of erythrocytes and lower hemoglobin concentration in the blood [15].

In the overall sample, the blood iron concentration predicted the potential of developing anemia. Because the protein hepcidin is the key regulator of the enteral iron availability, we assume that this protein in particular may have affected the enteral iron resorption [43]. Iron uptake and inflammation increase the concentration of hepcidin, which reduces the concentration of iron in the blood. Hepcidin is a 25 amino acid protein. It is found in humans and animals. It regulates and slows down iron absorption from the gut, placenta and reticuloendothelial system. Although it was not possible to obtain blood hepcidin levels from our patients, we hypothesize that there is an interaction between the availability of iron and protein. Analyses based on the overall sample are consistent with this interpretation. The negative relationship between the serum albumin and the hemoglobin concentration in the anemic subsample suggests that diminished nutrient availability impairs the erythro- and hematopoiesis of malnourished patients.

Our interpretations are supported by previous studies that suggested that albumin and hemoglobin associations might be explainable by the presence of the increased nutritional risk and malnutrition [3, 4]. In this vein, it has been argued that malnutrition (and consequently a reduced bioavailability of protein) leads to a reduced protein synthesis including albumin- and hemoglobin synthesis [13, 44, 45] which may in turn affect the hematopoiesis [4, 46–49]. This interpretation is consistent with observations of protein malnutrition leading to pathophysiological transformations in the bone and blood production defects in mice [46]. Similar observations have been made in human samples where it has been shown that anemic, malnourished geriatric patients run an elevated risk for developing hypoalbuminemia (i.e., so-called protein-deficiency anemia) [50, 51].

In our overall sample, serum free iron was the strongest predictor of the hemoglobin concentration. This finding was unexpected, because no further iron metabolism parameters differed between the groups. Because iron metabolism and chronic inflammation interact through the protein hepcidin it seems reasonable to assume that chronic inflammation plays a role in anemia development [43]. This is consistent with the pathophysiological perspective where anemia that has been caused by a functional lack of iron is called chronic inflammatory anemia [37]. Triggered by a chronic, subclinical inflammation, a disrupted iron balance manifests itself by a functional iron shortage [37, 38]. These potential trigger factors show complex interactions, especially when considering that a possible chronic inflammation could also play a central role in the pathophysiology of illness-associated malnutrition [49].

In previous studies, this interaction has been described as “Malnutrition-Inflammation-Complex” [42, 47]. In this vein, high levels of inflammation markers and low levels of nutritional status markers were shown to represent independent and significant predictors of a weak reaction to erythropoiesis-stimulators [14]. Recent reports contributed towards further clarifying these relationships, identifying mTOR (mechanistic target of rapamycin; a protein functioning as an enzyme in nutrient signaling pathways) as an important facilitator of this link [52–54]. mTOR coordinates protein translation with nutrient availability in organisms ranging from yeasts to humans. Moreover, this protein regulates the metabolic network in erythroid cells, thus controlling their balanced production and maturation [54, 55]. Furthermore, mTOR integrates the four prime regulatory inputs in erythroid cells: nutrients, growth factors, energy status, and signaling network stress [56, 57]. mTOR thus coordinates iron and protein availability with the cells` biochemical response. While few studies have thus far directly examined mTOR`s role in the red blood cells, it has been shown that red blood cells contain high levels of mTOR [9]. Consequently, it was suggested that the mTOR pathway is involved in the pathogenesis of anemia, particularly because it relates iron availability to cell growth and hemoglobin synthesis during erythropoiesis. Furthermore, red blood cells must balance the rate of globin synthesis with iron availability, so that free globin peptides will not aggregate in the absence of heme. Therefore, mTOR fine-tunes the balance between heme and globin levels in red blood cells [9]. The described biological makeup of these processes is consistent with our findings that nutritional risk plays a more important role in anemia onset than previously known.

### Strengths and limitations

As the study was cross-sectional and captures data at a single point in time, it does not account for temporal changes or the directionality of observed associations. Furthermore, the data collection was limited to only two centers, which constrains the generalizability of the results to a broader population. Conversely, the two aforementioned centers provide care for a specific group, namely geriatric patients who not only need assistance and general care, but also medical care on a daily basis. Consequently, the findings of this study are of particular relevance to this specific group of individuals and can inform the development of targeted healthcare strategies. Our research has considerable practical implications, with the potential to enhance the quality of care provided to geriatric patients.

One further strength of the study is the use of the GNRI, which offers a multifaceted method for assessing the nutritional risk of older adults. However, the GNRI is not sufficient on its own to clinically diagnose malnutrition. Future studies should aim to apply the full GLIM framework in combination with biochemical markers and clinical indicators to provide a more comprehensive understanding of how malnutrition contributes to anemia in older adults. It should also be noted that we did not apply corrections for multiple testing in the exploratory phase. This may increase the chance of false positives, and therefore, results from the univariate analyses should be interpreted as hypothesis-generating rather than confirmatory.

## Conclusions

Our results indicate that nutritional risk, particularly reduced protein status as indicated by serum albumin levels, is associated with anemia in hospitalized geriatric patients. While both anemic and non-anemic groups showed signs of elevated nutritional risk, anemic patients exhibited significantly lower GNRI scores and albumin concentrations, highlighting a stronger association between nutritional risk and anemia. Importantly, in the anemic subgroup, hemoglobin levels were significantly associated with both hematocrit and serum albumin concentration, whereas in the total sample and non-anemic subgroup, hemoglobin was primarily associated with hematocrit and serum iron. These findings emphasize that protein-related nutritional status plays a more prominent role in anemic individuals, rather than being a universal predictor across all older patients.

While our study design precludes causal inference, the results underscore the need to consider nutritional risk– especially protein deficiency– as a contributing factor to anemia in geriatric inpatient settings. Future longitudinal studies are warranted to explore these relationships more deeply and assess whether nutritional interventions targeting protein and iron intake can effectively prevent or mitigate anemia in geriatric patients.

## Data Availability

The data underlying this article are available in the Open Science Framework (OSF), at https://doi.org/10.17605/OSF.IO/RQZND.

## Abbreviations

BMI: Body mass index
CI: Confidence Interval
CRP: C-reactive protein
GLIM: Global Leadership Initiative on Malnutrition
GNRI: Geriatric Nutritional Risk Index
MCH: Mean corpuscular hemoglobin
MCHC: Mean corpuscular hemoglobin concentration
MCV: Mean Corpuscular Volume
mTOR: Mechanistic target of rapamycin
SD: Standard deviation
SE: Standard Error
VIF: Variance inflation factor
WHO: World Health Organization
